# Draft Genome Sequences of Four *Salmonella enterica* Strains Isolated from Turkey-Associated Sources

**DOI:** 10.1128/genomeA.01122-16

**Published:** 2016-10-13

**Authors:** Bijay K. Khajanchi, Jing Han, Kuppan Gokulan, Shaohua Zhao, Allen Gies, Steven L. Foley

**Affiliations:** a U.S. Food and Drug Administration, National Center for Toxicological Research, Jefferson, Arkansas, USA; b U.S. Food and Drug Administration, Center for Veterinary Medicine, Laurel, Maryland, USA; c Department of Microbiology and Immunology, University of Arkansas for Medical Sciences, Little Rock, Arkansas, USA

## Abstract

We report the draft genomes of four *Salmonella enterica* isolates evaluated for the contribution of plasmids to virulence. Strains SE163A, SE696A, and SE710A carry plasmids demonstrated to facilitate plasmid-associated virulence, while SE819 is less virulent and has been used as a recipient for conjugation experiments to assess plasmid-encoded virulence mechanisms.

## GENOME ANNOUNCEMENT

*Salmonella enterica* has been identified as the source of multiple outbreaks associated with meat and poultry products over the last few years in the United States and other countries (1). In the United States alone, it is estimated that more than 1 million *Salmonella* infections occur annually, resulting in nearly 20,000 hospitalizations and 400 deaths (2). Some *S. enterica* serovars cause more invasive infections than others; for example, a previous study demonstrated that the Heidelberg and Typhimurium strains were more invasive, responsible for 13% and 6% of infections, respectively, compared to the other serovars (3). Many *Salmonella* strains carry plasmids that harbor genes that contribute to increased virulence and antimicrobial resistance (4, 5). Previous studies in our laboratory characterized the impact of plasmids in *S. enterica* on antimicrobial resistance and virulence (4, 5).

We sequenced four *S. enterica* strains designated SE163A, SE696A, SE710A, and SE819, isolated from turkey-associated sources. SE163A and SE696A are virulent strains that possess plasmids including incompatibility group (Inc) FIB, IncA/C, and IncX4 (VirB/D4 type 4 secretion system–encoding plasmid) that carry virulence and/or antimicrobial resistance-associated genetic factors (5, 6). SE819 is a less virulent strain that lacks these virulence and antimicrobial resistance plasmids and has served as a recipient strain for studies evaluating the contribution of plasmids to antimicrobial resistance and virulence (4, 6, 7). Studies have demonstrated that genetic factors encoded by these transmissible SE163A- and SE696A-associated plasmids contribute to antibiotic resistance and the virulence in *Salmonella* species (6–8). SE710A also contains IncFIB, IncA/C, and IncX4 plasmids; however, contribution of these plasmids in virulence has not been examined for this isolate. Genome analysis of these particular *S. enterica* strains will be beneficial to our future understanding of the role of plasmid-encoded factors that contribute to virulence and antibiotic resistance.

Genomic DNA was extracted using a DNeasy blood and tissue kit (Qiagen, Valencia, CA, USA) and sequenced by the DNA Sequencing Core Facility at the University of Arkansas for Medical Sciences (UAMS; Little Rock, AR, USA). The DNA library was constructed with a Nextera XT DNA sample prep kit according to the manufacturer’s protocol (Illumina, San Diego, CA, USA), and sequencing was performed using the Illumina MiSeq with 2 × 250 paired-end reads. CLC Genomics Workbench version 8.5.1 (Qiagen, Germantown, MD, USA) was used for the trimming and *de-novo* assembly of the paired-end reads.

Draft genomes of these four *S. enterica* strains were annotated using the Rapid Annotation using Subsystem Technology (RAST) server (9), the Pathosystems Resource Integration Center (PATRIC) (10), and the NCBI Prokaryotic Genome Annotation Pipeline (PGAP) (Table 1). The G+C content for these strains is identical, with an estimate of 52.1%. Annotation using PATRIC (10) revealed that strain SE163A contains 5,448 coding sequences, in which 4,637 encode functional proteins, while 811 encode hypothetical proteins. Strain SE696A contains 5,296 coding sequences, in which 4,584 encode functional proteins and 712 encode hypothetical proteins. Strain SE710A possesses 5,337 coding sequences, in which 4,626 proteins have functional assignments and 711 encode as hypothetical. Strain SE819 contains 5,103 coding regions, in which 4,450 encode functional proteins and 653 are assigned as hypothetical proteins. 

**TABLE 1:** Summary of the genome sequence analysis of four *Salmonella enterica* strains isolated from turkey-associated sources

Strain	Location, yr	No. of contigs	Assembly size (bp)	G+C content (%)	No. of rRNAs	No. of tRNAs	Accession no.
SE163A	Ohio, 2002	257	5,202,941	52.1	7	74	LSZD00000000
SE696A	Midwest United States, 2000	230	5,096,557	52.1	16	78	LXHA00000000
SE710A	North Dakota, 1992	318	5,100,225	52.1	8	78	LXGZ00000000
SE819	Maryland, 2002	233	4,914,824	52.1	11	82	LSZE00000000

### Accession number(s).

The genome sequences of SE163A, SE696A, SE710A, and SE819 were deposited in GenBank under the accession numbers shown in Table 1.
